# Therapeutic Interventions to Manage Oral Mucositis and Their Impact on Quality of Life in Cancer Patients: An Umbrella Review

**DOI:** 10.1155/prm/3601001

**Published:** 2026-02-03

**Authors:** Joatan Lucas de Sousa Gomes Costa, Leonardo Díaz, Alain Chaple Gil, Gonzalo Rodríguez Martínez, Rodrigo Cabello Ibacache, Cristian Bersezio Miranda, Alfredo Von Marttens, Javier Basualdo, Eduardo Fernández Godoy, Milton Carlos Kuga

**Affiliations:** ^1^ Instituto de Ciencias Biomédicas, Universidad Autónoma de Chile, Santiago, Chile, uautonoma.cl; ^2^ Department of Prosthodontics, Faculty of Dentistry, University of Chile, Santiago, Chile, uchile.cl; ^3^ Department of Stomatology, Faculty of Dentistry, Universidad de Sevilla, Sevilla, Spain, us.es; ^4^ Perioplastic Institute, Santiago, Chile; ^5^ Facultad de Ciencias de la Salud, Universidad Autónoma de Chile, Santiago, Chile, uautonoma.cl; ^6^ Restorative Dentistry Department, Faculty of Dentistry, Dental School, University of Chile, Santiago, Chile, uchile.cl; ^7^ Graduate School, Faculty of Dentistry, University of Chile, Santiago, Chile, uchile.cl; ^8^ Department of Restorative Dentistry, School of Dentistry, São Paulo State University (UNESP), Araraquara, São Paulo, Brazil, unesp.br

**Keywords:** cancer treatment, gabapentin, oral mucositis, patient-reported outcomes, photobiomodulation, quality of life, supportive care, umbrella review

## Abstract

**Background:**

Oral mucositis (OM) is a debilitating complication of cancer therapy, particularly in head and neck cancer patients, with significant adverse effects on quality of life (QoL). Although numerous interventions have been investigated, their impact on QoL remains inconsistently reported and poorly synthesized.

**Methods:**

An umbrella review was conducted following PRISMA 2020 and Joanna Briggs Institute (JBI) guidelines. Five databases (PubMed, Embase, Scopus, Web of Science, and Cochrane Library) were searched from inception to March 2024. Reviews were eligible if they evaluated any therapeutic intervention for OM and reported QoL‐related outcomes using validated tools. Methodological quality was appraised using AMSTAR 2, and findings were narratively synthesized and thematically categorized. The protocol was registered in PROSPERO (CRD420251044088).

**Results:**

Eight systematic reviews (257 primary studies) met the inclusion criteria. Photobiomodulation, honey, black mulberry, and botanical agents such as SAMITAL showed consistent improvement in QoL domains, including pain relief, oral function, and emotional well‐being. Gabapentin demonstrated potential in reducing opioid use and early mucositis‐related pain, though findings were mixed across trials. QoL was most commonly assessed using the EORTC QLQ‐C30, FACT‐HN, and UW‐QOL. Overall, heterogeneity in intervention protocols and QoL instruments limited comparability.

**Conclusions:**

This review highlights the most promising interventions for improving QoL in patients with OM. Standardizing QoL measurement and prioritizing patient‐reported outcomes in future trials is essential to inform evidence‐based supportive oncology care.

## 1. Introduction

Oral mucositis (OM) is among the most frequent and debilitating complications experienced by cancer patients undergoing chemotherapy and/or radiotherapy, especially in head and neck malignancies [1]. OM is marked by erythema, ulceration, pain, dysphagia, and susceptibility to secondary infections, resulting from cytotoxic injury to the rapidly dividing oral epithelium. Although historically viewed as a localized inflammatory reaction, it is now understood to have broader systemic consequences, including nutritional decline, unplanned treatment interruptions, and a substantial reduction in patients’ quality of life (QoL). OM affects up to 80% of individuals receiving head and neck radiotherapy and about 40% of those undergoing standard chemotherapy [2]. In severe cases, OM can necessitate opioid analgesics, enteral feeding, or hospitalization, compromising the continuity of cancer care and increasing healthcare costs [3].

QoL has become a central outcome in oncology, especially in supportive and palliative care, reflecting its importance beyond traditional clinical measures. According to the World Health Organization (WHO), QoL encompasses an individual’s perception of their overall position in life within the context of their culture, values, goals, expectations, and concerns [4]. QoL is often severely reduced during aggressive cancer therapies. Toxicities such as OM exacerbate this decline, not only causing pain, ulceration, dysphagia, and infection but also leading to nutritional compromise, treatment interruptions, and substantial psychological distress. Given that up to 80% of patients receiving head and neck radiotherapy and 40% undergoing chemotherapy develop OM, interventions that effectively reduce treatment‐related toxicity without compromising oncologic efficacy are essential for improving patient‐centered outcomes [5].

Although QoL is increasingly used as a clinical endpoint in oncology trials, its measurement in OM‐focused studies has been inconsistent. Most primary studies evaluating OM interventions prioritize lesion severity, duration, and pain intensity, while patient‐reported outcomes (PROs) such as emotional well‐being, oral function, or global QoL receive less emphasis [6]. Furthermore, there is heterogeneity in the instruments used to measure QoL, with tools like the EORTC QLQ‐C30, UW‐QOL, and FACT‐H&N applied variably across studies. This lack of standardization complicates the synthesis of evidence and limits the clinical translation of findings [7].

In the past decade, a variety of pharmacological and nonpharmacological interventions have been investigated for the prevention and treatment of OM [8]. Among them, photobiomodulation therapy (PBMT) [9], honey [10], herbal agents (such as chamomile or black mulberry) [11], and combined oral care protocols have gained prominence due to their noninvasive nature and reported analgesic, anti‐inflammatory, or wound‐healing properties. Notably, several of these interventions are derived from complementary and integrative medicine paradigms, raising questions about their scientific validation and the robustness of supporting evidence. Nonetheless, patient preference and acceptance of these low‐risk interventions remain high, particularly when conventional pharmacologic options carry systemic side effects or limited efficacy [6].

Despite the proliferation of clinical trials and systematic reviews addressing OM interventions, the evidence concerning their impact on QoL remains fragmented. Some reviews focus exclusively on lesion‐based outcomes [12, 13], while others include QoL as a secondary endpoint without a detailed synthesis. In parallel, new clinical guidelines by professional bodies such as the Multinational Association of Supportive Care in Cancer (MASCC)/ISOO and the European Society for Medical Oncology (ESMO) are increasingly incorporating QoL considerations into supportive care recommendations. As such, there is a pressing need to consolidate and critically appraise the available evidence from systematic reviews that specifically report on QoL outcomes in patients undergoing OM‐related interventions during cancer therapy.

An umbrella review (UR) synthesizes evidence from multiple systematic reviews to provide a high‐level overview of a broad research area. Unlike traditional systematic reviews, it allows comparison of intervention effectiveness across diverse populations and outcomes, supporting evidence‐informed decisions and guideline development. In the context of OM and QoL, a UR helps identify which interventions most consistently improve PROs, highlight evidence gaps, and assess the methodological quality of existing reviews.

Therefore, the present UR aims to synthesize the best available evidence on the impact of therapeutic interventions for OM on QoL in cancer patients receiving active treatment. Specifically, we sought to (1) identify interventions with the strongest evidence for improving QoL in OM patients, (2) classify the affected QoL domains (e.g., physical, emotional, social, functional), and (3) assess the methodological quality and consistency of the systematic reviews included. In doing so, we aim to provide clinicians, researchers, and policymakers with a clear and evidence‐based understanding of how supportive OM interventions affect the lived experience of cancer patients.

To date, no prior UR has systematically integrated QoL‐specific findings across OM intervention studies, despite the well‐documented physical and psychological burden of this condition. Our review is therefore both timely and relevant, as QoL considerations are increasingly emphasized in personalized oncology care, survivorship planning, and health technology assessment. By adopting a rigorous methodology based on Preferred Reporting Items for Systematic reviews and Meta‐Analyses (PRISMA) [14] and the Joanna Briggs Institute (JBI) guidance [15], and using “A Measurement Tool to Assess Systematic Reviews 2” (AMSTAR 2) [16] for methodological quality appraisal, we ensure the robustness and transparency of our synthesis. Furthermore, by including a diverse range of interventions from high‐technology devices like PBMT to low‐cost agents such as honey, we capture the full spectrum of therapeutic possibilities relevant to both high‐income and resource‐constrained settings. Ultimately, understanding the impact of OM interventions on QoL is not only of clinical importance but also of ethical relevance. As cancer care shifts toward precision and patient‐centered models [17], the alleviation of treatment‐related suffering becomes a core therapeutic goal.

## 2. Methodology

### 2.1. Review Design

This UR was conducted to synthesize existing systematic reviews and meta‐analyses that examined the effectiveness of therapeutic interventions for OM on QoL outcomes in cancer patients undergoing active treatment. The study design followed the guidance of the JBI Manual for Evidence Synthesis and adhered to the PRISMA 2020 guidelines [18]. This UR was registered in the International Prospective Register of Systematic Reviews (PROSPERO; registration number: CRD420251044088). Because this URs synthesized data from previously published studies and did not involve direct contact with human participants, formal ethics committee approval and informed consent were not required.

As per JBI definitions [15], a UR integrates evidence from multiple systematic reviews addressing a broad clinical issue, providing a comprehensive summary for decision‐making. The present work employed a narrative synthesis approach, complemented by structured data extraction and quality appraisal, to summarize the effects of OM interventions on QoL‐related outcomes. We restricted inclusion to systematic reviews and meta‐analyses rather than individual randomized controlled trials (RCTs) to preserve the umbrella‐level design, avoid duplicating existing high‐quality syntheses, and provide decision‐makers with a concise summary of convergent evidence across heterogeneous interventions and populations. High‐quality RCT meta‐analyses not embedded within a systematic review were considered only if they fulfilled standard systematic review criteria, but none met our prespecified eligibility thresholds.

### 2.2. Search Strategy

A systematic and comprehensive search was conducted in five electronic databases: PubMed, Embase, Scopus, Web of Science, and the Cochrane Library. The search was limited to English‐language publications and included records from database inception to March 30, 2024. The search strategy combined terms related to “oral mucositis,” “cancer,” “quality of life,” and “systematic review” using both MeSH terms and free‐text keywords.

The search strategy was adapted for each database, and all records were exported into the Rayyan software for screening and deduplication. The complete search strings for all databases (PubMed, Embase, Scopus, Web of Science, and Cochrane Library) are provided in Supporting Table S1.

### 2.3. Eligibility Criteria

The inclusion criteria were based on the Patient/Population, Intervention, Comparison, and Outcome (PICO) framework and defined as follows:

• Population (P): Adults (≥ 18 years) undergoing active cancer treatment (chemotherapy, radiotherapy, or chemoradiotherapy) who developed or were at risk of developing OM.
• Intervention (I): Any therapeutic intervention (e.g., photobiomodulation, honey, herbal products, cryotherapy, oral care protocols) designed to prevent or treat OM.
• Comparison (C): Standard care, placebo, or no treatment.
• Outcome (O): Primary outcome: QoL (any domain or global score); secondary outcomes: pain, oral function, adherence to cancer treatment.

Only systematic reviews and meta‐analyses of RCTs or mixed designs that explicitly reported QoL data were included. Reviews that focused solely on lesion severity without PROs were excluded. Studies not written in English, reviews with overlapping datasets without added synthesis value, and narrative reviews were also excluded.

### 2.4. Study Selection

Two reviewers independently screened (AC and LD) titles and abstracts for relevance using the blinded feature in Rayyan. Full texts were obtained for all potentially eligible articles. Discrepancies in eligibility decisions were resolved through discussion, and a third reviewer was consulted when consensus was not achieved.

**Figure 1:**
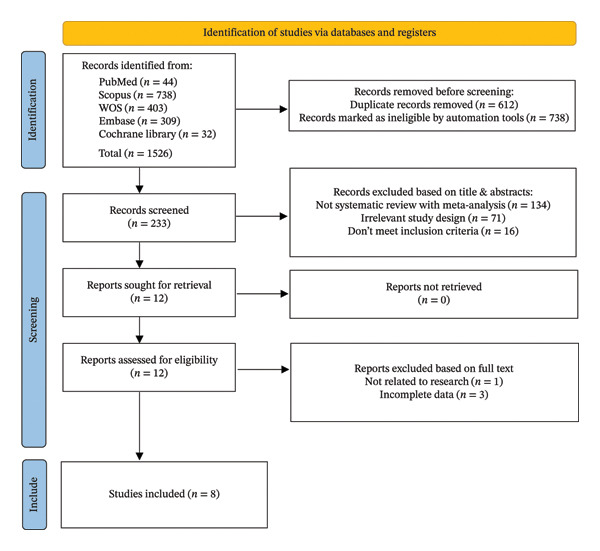
Flowchart PRISM.

The PRISMA 2020 flow diagram was used (Figure 1) to illustrate the process of study selection, including the number of records identified, screened, excluded, and finally included in the UR.

### 2.5. Data Extraction

A structured data extraction form was developed in Microsoft Excel and pilot‐tested for consistency. For each included review, we extracted the following information:

• Review authors and publication year
• Number and type of studies included
• Sample size and cancer type
• Type and delivery of intervention
• QoL measurement tools used (e.g., EORTC QLQ‐C30, FACT‐H&N, UW‐QOL)
• Direction and significance of QoL effects
• Adverse events and treatment adherence
• AMSTAR 2 quality rating

Where necessary, corresponding authors were contacted for clarification or to retrieve missing data. Extracted information was cross‐verified by two independent reviewers.

### 2.6. Risk‐of‐Bias Assessment

The methodological quality of each included systematic review was assessed using the AMSTAR 2 [16] checklist. This tool comprises 16 items and evaluates critical domains such as protocol registration, comprehensive literature search, justification for study exclusions, risk‐of‐bias assessment, appropriateness of meta‐analytical methods, and consideration of publication bias.

Two reviewers independently rated each review and categorized the overall confidence in results as high, moderate, low, or critically low, based on AMSTAR 2 criteria. Disagreements were resolved through consensus.

### 2.7. Data Synthesis

Given the heterogeneity in interventions, populations, and QoL measurement tools, a quantitative meta‐analysis was not feasible. Instead, a narrative synthesis approach was employed. The results were grouped thematically by intervention type, and QoL domains were mapped across studies.

We specifically reported whether the interventions had a positive, negative, or null effect on QoL, and noted which domains (e.g., pain, function, psychological well‐being, social functioning) were most improved. Where available, effect sizes, confidence intervals, and statistical significance were reported.

A cross‐tabulated evidence matrix was developed to display the QoL domains most frequently affected by each intervention type, allowing for visual comparison of therapeutic efficacy across outcomes. In addition, a summary of clinical recommendations based on the strength of evidence was generated.

Two reviewers independently coded the extracted data using an inductive thematic approach. Interventions were first grouped by type (e.g., photobiomodulation, honey, mulberry, chamomile, multiagent botanical formulations, pharmacologic analgesia, nonpharmacologic support). Within each group, outcomes were coded according to QoL domains (pain, oral function, emotional well‐being, social functioning, global QoL). Discrepancies in coding were resolved through discussion, and a third reviewer arbitrated when consensus was not reached. The final thematic map was used to structure the narrative synthesis and populate the cross‐tabulated evidence matrix (Table 1).

**Table 1:** Summary of QoL domains affected across interventions.

Intervention	Pain relief	Oral function	Emotional well‐being	Social functioning	Global QoL score
PBMT	✓✓✓	✓✓	✓	✓	✓✓✓
Honey	✓✓✓	✓	—	—	✓✓
Chamomile (aromatherapy)	✓✓	—	✓✓	✓	✓
Gabapentin	✓✓	✓	✓	—	✓
Black Mulberry	✓✓✓	✓✓	✓	—	✓✓
SAMITAL/FITOPROT	✓✓✓	✓✓	✓	✓	✓✓✓
Nonpharmacologic (nutrition)	✓	✓	—	—	—

*Note:* ✓✓✓ = consistent effect across multiple studies. ✓✓ = moderate evidence. ✓ = limited evidence. — not reported. PBMT, photobiomodulation therapy; SAMITAL, botanical multicomponent formulation; FITOPROT, phytotherapeutic formulation.

Abbreviation: QoL, quality of life.

When reviews reported discordant findings for the same intervention and outcome, we compared populations, intervention protocols, outcome measures, and methodological quality (AMSTAR 2) to identify potential sources of inconsistency. In the narrative synthesis, we gave greater weight to higher quality and more recent reviews and explicitly reported where evidence was conflicting.

During data extraction, we mapped primary RCTs across reviews to identify overlap. When multiple systematic reviews synthesized the same primary trials, we avoided double‐counting by (i) prioritizing the most comprehensive and methodologically robust review (based on AMSTAR 2), and (ii) summarizing overlapping evidence qualitatively rather than aggregating numerical results across reviews. Overlapping primary studies were noted in our evidence matrix and discussed narratively to contextualize apparent redundancies or discrepancies.

### 2.8. Patient and Public Involvement (PPI)

No patients or members of the public were involved in the design or conduct of this UR.

## 3. Results

### 3.1. Study Selection and Overview

A total of 786 records were identified across five electronic databases. After removing duplicates and screening titles and abstracts, 29 systematic reviews underwent full‐text evaluation. Eight reviews met all inclusion criteria and were included in this UR. The PRISMA flow diagram (Figure 1) summarizes the selection process.

These eight systematic reviews were published between 2019 and 2024 and collectively synthesized evidence from 257 primary studies involving adult cancer patients undergoing chemotherapy, radiotherapy, or both. All reviews evaluated the effects of therapeutic interventions for OM on QoL, either as a primary or secondary outcome. A summary of the reviews, interventions, and QoL outcomes is presented in Table 2.

**Table 2:** Comparison of therapeutic interventions for oral mucositis and their impact on quality of life (QoL).

Intervention	Study reference	QoL improvement	Additional benefits
Photobiomodulation (PBMT)	Potrich et al.[19]	Yes (significant improvement across all included studies)	Reduces mucositis severity (predominantly Grades 1–2), preserves treatment adherence
Honey	Liu et al. [20]	Yes (notable post‐treatment QoL improvements)	Decreases mucositis grade, alleviates pain, accelerates healing
Chamomile	Maleki et al. [21]	Mixed (improved with aromatherapy, not with syrup)	Reduces mucositis severity, anxiety, and depression; effectiveness varies by delivery route
Black mulberry formulations	Sindhe et al.[22]	Yes (QoL improved with reduced pain and OM severity)	Enhances appetite, weight maintenance, and reduces the severity and pain
Natural products (incl. FITOPROT and SAMITAL)	Zhang et al. [23]	Yes (notable improvements with agents such as FITOPROT)	Reduces OM grade and pain; alleviates xerostomia, dysphagia, and other treatment‐related complications
Nonpharmacological interventions (nutrition, LLLT)	Jin et al. [24]	No significant effect reported on QoL	Improved nutritional status; reduced incidence of oral and gastrointestinal mucositis during radiotherapy
PBMT with validated QoL instruments	Sánchez‐Martos et al. [25]	Yes (QoL improved in several RCTs using EORTC, UW‐QOL)	Reduced OM severity, shorter symptom duration, improved functional scores
Gabapentin	Baig et al. [26]	Mixed (significant in 1 RCT, positive trend in 2, no effect in 2)	Reduction or delay in opioid usage; early pain control; manageable side effects (e.g., somnolence, nausea); potential improvement in swallowing and systemic symptoms

*Note:* The following abbreviations are used in this table: PBMT: photobiomodulation therapy; EORTC: European Organization for Research and Treatment of Cancer QoL questionnaires (QLQ‐C30, QLQ‐HN35); UW‐QOL: University of Washington Quality of Life questionnaire; LLLT: low‐level laser therapy (historical term; replaced by PBMT); FITOPROT: proprietary phytotherapeutic formulation; SAMITAL: botanical multicomponent formulation for mucositis management.

Abbreviations: OM, oral mucositis; QoL, quality of life; RCT, randomized controlled trial.

### 3.2. Characteristics of Included Reviews

As summarized in Table 3, the included reviews addressed a range of interventions, including PBMT, natural products such as honey, chamomile, and black mulberry, and nonpharmacological strategies like nutritional support and oral hygiene protocols. Most studies focused on patients with head and neck cancer undergoing radiotherapy, but some included mixed oncological populations.

**Table 3:** Summary of study characteristics and main quality of life (QoL) outcomes.

Study reference	Intervention	Population	Study design	QoL assessment tool	Key QoL findings
Potrich et al. [19]	PBMT (various protocols)	Head and neck cancer (HNC) patients receiving radiotherapy	Systematic review (7 RCTs)	EORTC QLQ‐C30, UW‐QOL	Improved QoL associated with reduced severity of oral mucositis (mostly Grades 1‐2); enhanced RT tolerance
Liu et al. [20]	Honey	Cancer patients undergoing RT/CT	Meta‐analysis (19 RCTs)	Not consistently reported	Notable post‐treatment QoL improvement; associated with pain relief and mucositis severity reduction
Maleki et al. [21]	Chamomile (various forms)	Cancer patients (various types)	Systematic review (18 trials)	UW‐QOL, VAS	QoL benefits observed with aromatherapy; oral syrup less effective; also improved anxiety and depression
Sindhe et al. [22]	Black mulberry formulations	Patients undergoing RT or CT	Systematic review (4 trials)	EORTC QLQ–H&N35	Enhanced QoL with reduced incidence of Grade 3 OM, better pain control, and less weight loss
Zhang et al. [23]	Natural products (e.g., FITOPROT, SAMITAL, aloe, honey)	Cancer patients undergoing RT	Systematic review (36 trials)	EORTC QLQ‐C30, UW‐QOL, VAS	Significant QoL improvement in studies using FITOPROT, SAMITAL, and mulberry‐based formulations
Jin et al. [24]	Nonpharmacologic interventions (nutrition, exercise, PBMT)	HNC patients undergoing RT	Systematic review (27 RCTs)	Not specified	No significant impact on QoL reported; benefits focused on nutritional status and mucositis prevention
Sánchez‐Martos et al. [25]	PBMT (focused on QoL)	HNC patients receiving CRT	Systematic review (10 RCTs)	EORTC QLQ‐C30, UW‐QOL, FACT–HN	Improved QoL confirmed in several RCTs using validated instruments; reduced duration and OM severity
Baig et al. [26]	Gabapentin (prophylactic, oral)	HNC patients undergoing CRT	Systematic review (5 RCTs)	EORTC QLQ‐C30, QLQ‐HN35, FACT–HN, VHNSSv2, PROMS, VAS	Significant improvement in 1 RCT; positive trend in 2; neutral effect in 2

*Note:* This table summarizes the characteristics of the included studies and their main quality‐of‐life (QoL) outcomes in patients with oral mucositis (OM). Photobiomodulation therapy (PBMT) was evaluated using validated QoL instruments such as the European Organization for Research and Treatment of Cancer questionnaires (EORTC QLQ‐C30 and QLQ–H&N35), the University of Washington Quality of Life questionnaire (UW‐QOL), the Functional Assessment of Cancer Therapy–Head and Neck (FACT–HN), the Vanderbilt Head and Neck Symptom Survey Version 2 (VHNSSv2), patient‐reported outcome measures (PROMS), and the visual analog scale (VAS). Other abbreviations include head and neck cancer (HNC), radiotherapy (RT), chemotherapy (CT), and chemoradiotherapy (CRT). Natural interventions such as FITOPROT and SAMITAL were also assessed for their impact on QoL across multiple domains.

QoL was assessed using a variety of validated tools, most commonly the EORTC QLQ‐C30, UW‐QOL, FACT‐H&N, and visual analog scales (VASs) for pain. The studies varied in methodological quality, reporting styles, and the extent to which QoL domains were analyzed.

### 3.3. Quality Assessment and Risk of Bias

According to the AMSTAR 2 appraisal (Supporting Table S2), the overall methodological quality of the included systematic reviews varied substantially, with most reviews exhibiting at least one critical or multiple noncritical weaknesses. Only two reviews (Potrich [19]; Sánchez‐Martos [25]) achieved a rating of “moderate confidence,” indicating relative methodological soundness, including appropriate risk‐of‐bias assessments, protocol registration, and comprehensive literature searches. In contrast, several reviews, including those by Jin [24] and Baig [26], were downgraded due to omissions in protocol reporting, lack of publication bias analysis, and insufficient handling of excluded studies. Notably, the majority of reviews did not perform meta‐analyses (indicated as “No MA” in the tool), which, although not penalized, reflects the heterogeneity of included studies and limits quantitative synthesis. The prevalence of critical flaws across multiple reviews underscores the urgent need for stricter adherence to review protocols and transparent reporting standards in future evidence syntheses focused on OM interventions.

### 3.4. Impact of Interventions on QoL

Where findings were inconsistent across reviews, we highlight these discrepancies and relate them to differences in QoL instruments, intervention protocols, and risk‐of‐bias profiles.

#### 3.4.1. PBMT

As shown in Tables 2 and 4, PBMT was consistently associated with significant improvements in QoL and reductions in OM severity. Potrich et al. [19] reported that PBMT significantly decreased the incidence of severe OM (from 48.3% in controls to 21.4% with PBMT; *p* < 0.01) and improved oral functional domains, including swallowing and oral pain, reflected in higher scores on the University of Washington Quality of Life Questionnaire (UW‐QOL v4) and better tolerance of radiotherapy sessions. Similarly, the systematic review by Sánchez‐Martos et al. [25], which included 10 RCTs and 759 patients, found that PBMT reduced mean pain scores (VAS reductions of 2–3 points in several trials), shortened the duration of OM, and improved QoL across instruments such as the European Organization for Research and Treatment of Cancer Quality of Life Questionnaire (EORTC QLQ‐C30), Quality of Life Questionnaire, Head and Neck 35 (QLQ‐HN35), University of Washington Quality of Life (UW‐QOL), and Functional Assessment of Cancer Therapy–Head & Neck (FACT–HN). Improvements consistently involved physical functioning, swallowing, saliva‐related discomfort, and global health status. Importantly, Sánchez‐Martos et al. also highlighted enhanced treatment adherence, with PBMT reducing the likelihood of chemoradiotherapy interruption due to painful OM. Together, these findings confirm that PBMT offers clinically meaningful benefits across multiple QoL and symptom domains.

**Table 4:** Primary outcomes, intervention protocols, effect sizes, and secondary outcomes in systematic reviews of oral mucositis.

Study reference	Primary outcome	Intervention details	Effect size or direction	Secondary outcomes
Potrich et al. [19]	OM severity, quality of life (QoL)	PBMT applied 5×/week prior to radiotherapy (632–830 nm; 2–12 J/cm^2^)	Reduced OM severity; QoL preserved vs control	Pain reduction, improved swallowing comfort; fewer treatment interruptions
Liu et al. [20]	OM grade, pain, QoL	Natural/raw honey rinses 3–4×/day; some trials used Manuka honey	WMD pain ≈ −3.25; RR for Grades 3‐4 OM≈0.18	Nutritional maintenance, reduced xerostomia, improved ability to eat, fewer RT interruptions
Maleki et al. [21]	OM reduction, QoL, anxiety	Chamomile as mouthwash, syrup, tea, cream, aromatherapy	Consistent OM reduction; QoL improvement mainly with aromatherapy	Anxiety reduction, better sleep, mild analgesic effect; improved mucosal hydration
Sindhe et al. [22]	OM grade, pain, QoL, weight loss	Black mulberry (syrup or molasses), 2–4×/day	Grade 3 OM: 26.8% ⟶ 2.7%; pain and weight outcomes improved	Dry mouth improvement, weight gain/less weight loss; reduced dysphagia
Zhang et al. [23]	OM grade, pain, xerostomia, QoL	20 natural products (honey, aloe, curcumin, plant extracts); various routes	Significant reduction in OM and symptoms; QoL improved in ∼75% studies	Xerostomia reduction, decreased dysphagia, antioxidant/anti‐inflammatory effects
Jin et al. [24]	OM occurrence, nutritional status, QoL	Diet support, cryotherapy, PBMT, oral care protocols	No significant QoL differences; OM prevention and nutritional benefits observed	Nutritional stabilization, reduced hospitalization, improved adherence to CRT
Sánchez‐Martos et al. [25]	OM grade, pain, QoL	Diode/He‐Ne laser (650–850 nm); 5×/week during CRT	Reduced OM grade and pain; QoL improvement significant	Shorter OM duration, fewer severe OM episodes, improved oral function
Baig et al. [26]	Pain severity, QoL, opioid use	Gabapentin 900–3600 mg/day during CRT	Significant pain reduction in 1 RCT; trend in 3; opioid use reduced	Reduced opioid consumption, better sleep, improved functional eating, fewer emergency visits

*Note:* The following abbreviations are used in this table: Measures of treatment effect include relative risk (RR) and weighted mean difference (WMD). Photobiomodulation parameters are expressed in nanometers (nm) for wavelength and joules per square centimeter (J/cm^2^) for energy density. Mucositis grading systems referenced include the World Health Organization (WHO) scale, the Radiation Therapy Oncology Group/European Organization for Research and Treatment of Cancer (RTOG/EORTC) scale, and the Common Terminology Criteria for Adverse Events (CTCAE). Botanical formulations mentioned include FITOPROT and SAMITAL.

#### 3.4.2. Natural Products: Honey, Chamomile, Mulberry

Honey was consistently effective in improving QoL, with Liu et al. [20] reporting a mean reduction of −3.25 points in VAS pain scores (95% CI −4.12 to −2.38) and a risk ratio of 0.18 (95% CI 0.10–0.32) for the development of severe OM (Grades 3–4), indicating an 82% relative risk reduction (Table 4). Honey also shortened OM duration by approximately 3.9 days (*p* < 0.01), and improved swallowing and oral comfort subscales on the European Organization for Research and Treatment of Cancer Quality of Life Questionnaire–Head and Neck 35 (EORTC QLQ–H&N35).

Black mulberry formulations demonstrated similarly robust effects. Sindhe et al. [22] reviewed four studies (*N* = 297) and found that the incidence of Grade 3 OM decreased from 26.8% in the control group to 2.7% in patients treated with mulberry molasses by Week 5 of radiotherapy. Improvements extended to QoL domains—patients receiving mulberry showed better EORTC QLQ–H&N35 global health scores, reduced pain (median VAS: 3 vs. 5 in controls at Week 6), and more favorable nutritional trajectories, including less weight loss (e.g., −3.3 kg vs. −8.0 kg with grape molasses at Week 6) and, in some trials, weight gain during chemotherapy cycles. Mulberry also reduced dry mouth severity by Radiation Therapy Oncology Group/European Organization for Research and Treatment of Cancer Questionnaire (RTOG/EORTC) scores from 2.18 ± 0.50 to 0.54 ± 0.03 by Week 3.

Chamomile showed mixed but noteworthy effects. In Maleki et al. [21], chamomile aromatherapy produced significant improvements in emotional well‐being, reduced mucositis‐related discomfort (*p* < 0.05), and modest improvements in VAS pain scores. However, oral chamomile formulations yielded inconsistent results across trials, with some studies showing no significant differences in OM severity or QoL (Tables 3 and 4). These discrepancies likely reflect heterogeneity in chamomile preparation, dosing frequency, and outcome measurement timing.

Overall, botanical therapies such as honey and black mulberry exhibit consistent and clinically relevant improvements in pain, OM severity, and QoL, whereas chamomile’s effects appear formulation‐dependent and less robust.

Zhang et al. [23] reported that 75% of the clinical trials evaluating natural agents showed improvements in QoL across at least one domain, with the strongest and most consistent effects observed for FITOPROT and SAMITAL. SAMITAL demonstrated significant superiority over placebo in the ROSAM (Role of SAMITAL in the Prevention and Treatment of Chemo‐Radiotherapy‐Induced Oral Mucositis in Head and Neck Carcinoma) Phase II trial, with improvements in global QoL, social eating, swallowing, and dry mouth domains on the EORTC QLQ‐C30 and QLQ‐H&N35 scales (overall *p* < 0.05). Reductions in symptom burden included better oral pain control and improved Oral Mucositis Assessment Scale (OMAS) and WHO mucositis scores. FITOPROT, evaluated in two RCTs, showed progressive reductions in mucositis severity between the 15th and 21st radiotherapy sessions (WHO: *p* = 0.03; National Cancer Institute’s Common Terminology Criteria for Adverse Events Scale (NCI‐CTCAE): *p* = 0.02), alongside improvements in QoL trajectories—particularly in oral discomfort, swallowing ability, and functional limitation although between‐group differences did not always reach statistical significance. The biological plausibility of these benefits is supported by FITOPROT’s demonstrated reduction in salivary nitrite levels (−15.2 μM from baseline vs. +9.7 μM in controls; *p* = 0.04) and downward trends in IL‐8 expression. Together, these findings suggest that botanical formulations such as FITOPROT and SAMITAL not only ameliorate mucositis severity but also yield multidimensional QoL benefits, likely mediated through improved oral function, reduced dysphagia, and enhanced daily communication and social engagement.

#### 3.4.3. Nonpharmacologic Interventions

Jin et al. [24] reviewed a broad range of nonpharmacological interventions including nutritional protocols, cryotherapy‐related symptom management, and oral hygiene–oriented care modalities as part of a large meta‐analysis of 27 RCTs involving 2736 patients with head and neck cancer undergoing radiotherapy. Although several interventions (particularly nutritional support, psychological counseling, and low‐level laser therapy) significantly reduced the incidence and severity of OM and improved nutritional indicators such as body weight, body mass index (BMI), and malnutrition risk, no significant improvements in overall QoL were observed. All included studies assessed QoL using the EORTC QLQ‐C30, and the pooled analysis demonstrated no meaningful difference between intervention and control groups (MD = 1.72; 95% CI: −1.02 to 4.46; *p* = 0.22; *I* ^2^ = 55%). The authors also highlighted a lack of standardized QoL measurement timepoints and substantial heterogeneity in how QoL outcomes were operationalized, contributing to inconsistent findings regarding PROs (see Table 4).

#### 3.4.4. Gabapentin

The systematic review by Baig et al. [26], which synthesized five RCTs, offers a more granular understanding of the prophylactic use of gabapentin for OM‐related pain in patients undergoing chemoradiotherapy for head and neck cancer. Quantitatively, the evidence shows heterogeneous but clinically relevant effects. For instance, Cook et al. [27] reported modest reductions in PROMS pain scores at 6 weeks post‐treatment, with mean mouth pain of 3.1 ± 2.2 in the gabapentin group versus 2.2 ± 1.9 in controls (*p* = 0.49), and swallowing‐related discomfort of 14.2 ± 9.4 versus 8.2 ± 6.7 (*p* = 0.10). Smith et al. demonstrated a statistically significant reduction in pain based on the VHNSSv2 pain subscale, where gabapentin produced lower end‐of‐treatment pain scores across percentiles; the adjusted mean difference favored gabapentin with *p* < 0.05, indicating cumulative analgesic benefit. In Sung Jan Ma et al., pain assessed via the EORTC QLQ‐HN35 yielded mean scores of 45 ± 23 in the gabapentin arm—lower than expected for this treatment population, although between‐group differences were not statistically significant.

Across studies, opioid‐related outcomes showed more consistent benefit. Herman et al. [28] reported that 42% of patients receiving high‐dose gabapentin (2700 mg/day) required no opioids, compared with 7% in the low‐dose/methadone arm (*p* = 0.002), and the median time to first opioid dose increased from 18.2 to 22.6 days (*p* = 0.09). Cook et al. [27] observed lower opioid exposure (gabapentin: 15.6 DME/day vs. placebo: 22.2 DME/day, *p* = 0.32), and in Ma et al. [29], 62.5% of patients treated with gabapentin alone did not require opioids during chemoradiation. QoL outcomes, measured through validated instruments including the EORTC QLQ‐C30, QLQ‐HN35, Functional Assessment of Cancer Therapy–Head & Neck (FACT‐HN), Vanderbilt Head & Neck Symptom Survey Version 2.0 (VHNSSv2), Patient‐Reported Outcome Measures (PROMS), and VAS, were significantly improved in only one RCT, neutral in two, and suggestive of a positive but nonsignificant trend in the remaining two. Notably, Herman et al. documented statistically significant improvements in global health status based on EORTC QLQ‐C30 (*p* < 0.05), while early‐treatment functional stability (baseline to Week 3) remained preserved (*p* = 0.92).

Despite variability, the consistent signals across trials including delayed opioid initiation, improvements in mucosal sensitivity, and lower pain trajectories in higher dose protocols highlight gabapentin’s neuromodulatory potential. Adverse events were generally mild, with no severe toxicities reported in two RCTs and predictable somnolence, nausea, or skin reactions in others. Collectively, these quantitative outcomes reinforce that gabapentin may confer clinically meaningful benefits in pain modulation and opioid sparing during chemoradiotherapy, even where statistical thresholds are not uniformly met.

### 3.5. Affected QoL Domains and Measurement Tools

Across reviews, the QoL domains most consistently impacted by interventions were pain relief, oral function, and emotional well‐being. As detailed in Table 1, PBMT and natural products like honey and SAMITAL consistently improved pain scores and eating ability. Chamomile aromatherapy improved emotional symptoms, including anxiety and sleep disturbances.

Measurement heterogeneity limited cross‐review comparability. Only four reviews explicitly mapped interventions to QoL domains using validated instruments. VAS was the most frequently used pain measure; UW‐QOL and FACT‐H&N were used in head and neck cancer populations, but minimally important differences (MIDs) were seldom reported.

### 3.6. Summary of Evidence Strength and Clinical Implications

The clinical utility of the interventions evaluated is summarized in Table 5. PBMT and honey demonstrated the most consistent evidence for QoL improvement, with strong recommendations for clinical use. Mulberry and FITOPROT showed promising results, though more trials are needed to confirm their efficacy. Chamomile’s benefit appears formulation‐dependent.

**Table 5:** Clinical recommendations based on systematic review evidence.

Intervention	Clinical context of use	Level of evidence	Recommendation strength
Photobiomodulation therapy (PBMT)	Prevention and treatment of oral mucositis (OM) in head and neck cancer during RT/CRT	Moderate to high (7 RCTs with consistent QoL and OM outcomes)	Strong recommendation for prevention and symptom control
Honey	Prophylaxis and symptomatic relief for OM in patients undergoing RT or CT	Moderate (19 RCTs; consistent pain and OM severity reductions)	Strong recommendation for adjunctive care in OM management
Chamomile	Adjunctive therapy for OM, anxiety, and mucosal healing in cancer patients	Low to moderate (18 trials; variable QoL and delivery modes)	Conditional recommendation depending on delivery form
Black mulberry	Management of OM and pain control during RT/CT in head and neck cancers	Moderate (4 trials with strong effect size and consistency)	Strong recommendation for OM symptom relief in RT/CT
Natural products (e.g., aloe, SAMITAL)	Integrative supportive care in OM prevention and symptom mitigation	Moderate (36 studies with varied quality and heterogeneity)	Conditional recommendation; agent‐specific effectiveness
Nonpharmacologic support (nutrition)	General supportive care during RT; not primarily focused on QoL improvement	Low (QoL not systematically assessed; mostly clinical outcomes)	Weak recommendation as adjunct only
PBMT with validated QoL tracking	Structured PBMT protocols applied during CRT with outcome monitoring	Moderate to high (10 RCTs; validated QoL outcomes improved)	Strong recommendation when QoL metrics are included
Gabapentin	Management of OM‐related pain and opioid reduction in CRT for HNC	Low to moderate (5 RCTs, variable outcomes)	Conditional recommendation; promising but inconsistent results

*Note:* The abbreviations used in this table include the following: photobiomodulation therapy (PBMT), radiotherapy (RT), chemotherapy (CT), chemoradiotherapy (CRT).

Abbreviations: HNC, head and neck cancer; OM, oral mucositis; QoL, quality of life; RCT, randomized controlled trial.

Nonpharmacologic interventions were associated with lower OM incidence but demonstrated weak QoL evidence. Overall, the UR supports the integration of PBMT and selecting natural agents into supportive care protocols for OM in cancer patients.

### 3.7. Population, Risk of Bias, Outcome Relevance, Protocol Adherence, and Statistical Robustness (PROPS) Framework Analysis

To facilitate a structured interpretation of the evidence, we applied the PROPS framework [30]. This analytical model supports the qualitative appraisal of heterogeneous systematic reviews and enhances transparency in umbrella‐level synthesis.

#### 3.7.1. Population

The included reviews primarily addressed adult cancer patients undergoing radiotherapy, chemotherapy, or chemoradiotherapy, with a focus on head and neck malignancies. PBMT reviews (Potrich [19]; Sánchez‐Martos [25]) and natural product reviews (Liu [20]; Zhang [23]) included large, clinically relevant populations with moderate‐to‐high OM risk. However, some reviews (e.g., Maleki [21]; Sindhe [22]) had narrower inclusion criteria and concentrated on specific cancer sites or regional practices (e.g., trials from Turkey or Iran), which may limit generalizability. Pediatric populations and patients in palliative care settings were not represented.

#### 3.7.2. Outcome Relevance

All reviews included reported QoL as either a primary or secondary outcome, yet few provided detailed domain‐specific analysis. In most cases, QoL was reported alongside pain, mucositis grade, or treatment duration, rather than as a standalone construct. Only the PBMT and FITOPROT reviews consistently link therapeutic effects to validated QoL instruments across multiple domains (e.g., pain, swallowing, emotional distress). Chamomile and nutritional interventions reported QoL inconsistently or via unvalidated measures. The most consistently affected domains were pain, eating, social functioning, and psychological well‐being (Table 1).

#### 3.7.3. Protocol Adherence

Protocol registration was not universally reported. Only three of the eight reviews clearly registered their protocol on PROSPERO or a comparable repository. Lack of transparency in review planning, especially concerning predefined outcome domains and inclusion/exclusion criteria, represents a limitation. Furthermore, just two reviews followed JBI or PRISMA standards comprehensively. The absence of predefined subgroup analyses and domain mapping impairs the interpretability of QoL‐specific findings.

#### 3.7.4. Statistical Robustness

Given the heterogeneity of interventions and QoL metrics, most reviews employed narrative synthesis rather than formal meta‐analysis. Where meta‐analytic pooling was attempted, it was limited to pain and mucositis severity outcomes, not multidimensional QoL. Confidence intervals and effect sizes were reported inconsistently, and MIDs were rarely mentioned. Consequently, while some interventions demonstrated statistically significant results, clinical significance remains uncertain for many.

#### 3.7.5. PROPS Analysis

This PROPS analysis reinforces that while PBMT and honey‐based interventions are supported by the most consistent and robust evidence, the broader field suffers from heterogeneity in design, reporting standards, and methodological transparency. These findings inform both clinical application and future trial design, emphasizing the need for protocol registration, standardized QoL measures, and rigorous statistical reporting. Table 6 highlights that protocol adherence and statistical robustness were the weakest domains across reviews, underscoring critical priorities for improving the quality and interpretability of future evidence syntheses (Table 6).

**TABLE 6:** PROPS‐based appraisal of included evidence syntheses on supportive interventions for oral mucositis in adult oncology patients, with emphasis on quality‐of‐life (QoL) reporting and methodological rigor.

PROPS domain	Observations	Appraisal
Population	Adults with cancer, mostly HNC under RT/CRT; few reviews include broader or diverse populations	Moderate to high
Risk of bias	Generally low to moderate; few assessments of publication bias or risk stratification	Moderate
Outcome relevance	QoL reported inconsistently; PBMT and SAMITAL show strong outcome relevance	Moderate to high
Protocol adherence	Only 3/8 reviews registered protocols; some lacked PRISMA/JBI adherence	Low to moderate
Statistical robustness	Limited meta‐analytical data for QoL; few MIDs or confidence intervals reported	Low to moderate

## 4. Discussion

This UR synthesized evidence from eight systematic reviews evaluating the impact of therapeutic interventions for OM on QoL in cancer patients undergoing active treatment. Our findings provide a comprehensive overview of current supportive care strategies, highlight the most effective interventions in terms of patient‐centered outcomes, and identify critical methodological limitations that hinder clinical translation. By integrating the evidence through the PROPS framework, we also offer a structured critique of the robustness and relevance of existing literature, aligned with the growing emphasis on QoL as a core outcome in oncology.

### 4.1. Principal Findings and Clinical Relevance

PBMT and natural products particularly honey, black mulberry, and SAMITAL demonstrated the strongest and most consistent evidence for improving QoL [19, 20, 22, 23, 25]. Improvements were noted across multiple domains, including pain relief, swallowing function, emotional well‐being, and treatment adherence. These findings underscore the evolving role of nonpharmacologic and complementary interventions in the supportive management of OM.

PBMT was associated with reduced mucositis severity and improvements in physical and emotional functioning [25]. These outcomes are especially relevant in head and neck cancer patients, where OM significantly interferes with nutrition, speech, and social functioning.

Honey, evaluated across 19 RCTs, reduced OM severity and pain while enhancing global QoL scores [20]. The consistency of findings and accessibility of this intervention make it a compelling option, especially in resource‐constrained settings.

Chamomile, examined by Maleki [21], yielded mixed results. Aromatherapy formulations improved QoL and psychological well‐being, whereas oral preparations (e.g., syrups and teas) showed inconsistent effects. Black mulberry, assessed in a review by Sindhe [22], was linked to significant reductions in OM severity and improved appetite, pain control, and QoL.

Zhang et al. [23] reviewed 36 trials on various natural products, including SAMITAL, FITOPROT, aloe vera, and turmeric. These agents were associated with improvements in pain, xerostomia, and global QoL scores, particularly in patients undergoing radiotherapy. Notably, SAMITAL and FITOPROT also showed benefits in domains related to social functioning, possibly reflecting improvements in speech clarity, eating comfort, and interpersonal communication—factors that play a critical role in daily social engagement during cancer treatment.

In contrast, Jin [24] synthesized data on nonpharmacologic strategies such as nutrition, oral hygiene, and low‐level laser therapy, which showed positive effects on OM prevention but minimal impact on QoL. The authors noted that QoL was seldom measured systematically or reported as a primary endpoint.

Finally, the review by Maleki et al. [21] captured the variability of botanical interventions. Chamomile’s efficacy was strongly dependent on the route of administration, frequency, and patient population.

An important finding of this review is the emerging evidence regarding the prophylactic use of gabapentin as a pharmacological intervention for OM pain management. Although the overall outcomes were mixed, the systematic review by Baig et al. [26] demonstrated a statistically significant reduction in pain and opioid consumption in at least one RCT, with a favorable trend observed in two others. QoL improvements were particularly evident in domains such as mucosal sensitivity, swallowing function, and emotional well‐being. These effects, coupled with a manageable side effect profile, suggest that gabapentin may serve as a valuable adjunct in supportive care protocols, particularly in clinical scenarios where opioid‐related adverse effects or dependency are of concern.

Beyond symptomatic relief, PBMT appears to modulate neuroinflammatory pathways, enhance mitochondrial function, and promote tissue repair through upregulation of growth factors and angiogenesis, which may translate into more sustained improvements in oral function and overall QoL [19]. Honey [20] and botanical formulations [21–23] combine topical anti‐inflammatory, antioxidant, and antimicrobial effects with mucosal coating and lubrication, reducing nociceptor activation and improving swallowing comfort. Gabapentin exerts central and peripheral neuromodulatory effects by binding to *α*2*δ* subunits of voltage‐gated calcium channels, dampening neuropathic and mucositis‐related pain and potentially reducing anxiety associated with anticipated oral discomfort [26]. These mechanisms collectively suggest that QoL gains may reflect not only reduced ulcer burden but also modulation of pain processing, sleep, and emotional distress.

### 4.2. Interpretation Through the PROPS Framework

The PROPS framework offered critical insight. While populations were mostly appropriate—adults undergoing radiotherapy or chemotherapy—there was a notable absence of pediatric or palliative patients, limiting generalizability.

Methodological quality, assessed using AMSTAR 2, was generally moderate. PBMT reviews (Potrich [19]; Sánchez‐Martos [25]) demonstrated good adherence to protocol transparency and risk‐of‐bias evaluation. Reviews by Liu [20] and Zhang [23] lacked formal GRADE assessments but were methodologically consistent.

Outcome relevance varied significantly. QoL was assessed using validated tools in PBMT and SAMITAL studies (Potrich [19]; Zhang [23]), whereas other reviews such as Jin [24] and Maleki [21] used heterogeneous or nonstandardized measures, limiting cross‐comparability.

Few reviews (e.g., Sindhe [22]; Sánchez‐Martos [25]) incorporated domain‐specific QoL analyses. Statistical robustness was limited across the dataset; only Liu [20] conducted a meta‐analysis with quantified effect sizes on QoL‐related endpoints.

Compared with prior narrative or scoping overviews on OM, which predominantly emphasized lesion‐based outcomes and incidence reduction, our UR extends the field by focusing specifically on QoL endpoints and PROs across interventions. Whereas earlier general reviews (e.g., Colella et al.; Shetty et al.) summarized preventive and therapeutic strategies, they rarely synthesized QoL domains or mapped interventions against validated QoL instruments. Our findings complement and deepen these overviews by identifying which interventions most consistently improve pain, oral function, and emotional well‐being and by appraising the QoL evidence through PROPS and AMSTAR 2 lenses.

### 4.3. Limitations of QoL Measurement Across Included Reviews

Despite growing consensus on the importance of PROs, our synthesis revealed considerable heterogeneity and underutilization of validated QoL instruments in OM‐related research. While some systematic reviews (e.g., Potrich; Zhang) [19, 23] employed standardized tools such as the EORTC QLQ‐C30 and FACT‐HN, many failed to report domain‐specific findings or MIDs, limiting the interpretability and clinical relevance of outcomes. VASs were frequently used to measure pain, yet rarely contextualized within broader QoL frameworks. Moreover, several reviews combined studies that used disparate measurement tools ranging from generic instruments to condition‐specific ones—without applying methodological safeguards such as sensitivity analysis or data standardization. Only a few reviews mapped intervention effects to specific QoL domains (e.g., swallowing, emotional well‐being), further complicating cross‐study comparability. This inconsistency highlights an urgent need for standardized outcome sets and a stronger emphasis on clinically meaningful change thresholds in future OM research. Publication bias is a relevant concern in this field. Only a minority of included reviews formally assessed publication bias (e.g., via funnel plots or statistical tests), and none incorporated gray literature or non‐English databases. Positive trials of novel or complementary interventions may therefore be over‐represented, while null or negative findings remain unpublished. Our restriction to English‐language systematic reviews further amplifies this limitation. Consequently, the apparent consistency of benefit observed for PBMT, honey, and certain botanicals should be interpreted with caution, acknowledging the potential underrepresentation of negative studies or non‐English evidence.

### 4.4. Clinical and Policy Implications

The results of this UR offer critical guidance for evidence‐based decision‐making in supportive oncology. Interventions such as PBMT, honey, and gabapentin demonstrate meaningful, patient‐centered benefits and should be considered for inclusion in updated clinical guidelines, including those by MASCC/ISOO and the ESMO. Moreover, these findings underscore the importance of integrating QoL metrics into routine oncology practice. QoL assessments should inform treatment planning, risk‐benefit discussions, and follow‐up strategies—particularly in head and neck cancer patients, where OM significantly affects nutritional status, social functioning, and mental health. By embedding validated QoL measures into shared decision‐making processes, clinicians can align therapeutic choices with patient preferences and improve overall treatment satisfaction. Lastly, from a health systems perspective, interventions with demonstrated QoL benefits, especially nonpharmacologic, low‐cost therapies such as honey or botanical formulations—may offer scalable, resource‐sensitive strategies that enhance value‐based cancer care. An additional practical advantage of PBMT over topical pharmacologic treatments (e.g., medicated mouthwashes or gels) is that PBMT is noncontact and does not typically provoke stinging or burning sensations on already ulcerated mucosa. This is particularly relevant in patients with extensive ulceration or heightened oral sensitivity, as well as in younger patients who may poorly tolerate repeated mouth rinses. Thus, PBMT may offer superior acceptability and adherence compared with some topical regimens, even when analgesic efficacy is similar.

Safety is a critical consideration when recommending PBMT in oncology settings. Consistent with the MASCC/ISOO position statement [31] and the WALT recommendations [32], we found no systematic signal of increased tumor recurrence, treatment interruption, or serious adverse events attributable to PBMT in the included reviews. Reported side effects were rare and generally mild (e.g., transient warmth or discomfort at the irradiation site). Nonetheless, strict adherence to evidence‐based PBMT parameters (wavelength, energy density, timing relative to RT/CRT) and avoidance of direct irradiation of tumor masses remain essential to minimize theoretical oncologic risks.

### 4.5. Considerations for QoL Interpretation and Instrument Standardization

#### 4.5.1. MIDs

A critical limitation identified across the included systematic reviews is the lack of reporting on MIDs for QoL instruments. MIDs are essential for interpreting whether statistically significant changes are also clinically meaningful to patients. Although few studies explicitly stated MID thresholds, literature suggests approximate MID values for the most common tools used in this review: for the EORTC QLQ‐C30, a change of ≥ 10 points on a 0–100 scale is generally considered clinically significant; for the FACT‐H&N, a difference of 6–12 points in the total score may represent a meaningful change; and for UW‐QOL, a ≥ 7–10 point change per domain is often cited as a relevant threshold. The absence of consistent MID use across reviews complicates comparative assessment and may lead to over‐ or underestimation of intervention effects. Future systematic reviews and clinical trials should explicitly incorporate MIDs to contextualize QoL improvements and support decision‐making aligned with patient priorities.

Where numerical data were available, we interpreted QoL changes in relation to published MID thresholds (e.g., ≥ 10 points for EORTC QLQ‐C30; 6–12 points for FACT‐H&N; ≥ 7–10 points per UW‐QOL domain). Interventions such as PBMT, honey, and SAMITAL frequently produced changes that exceeded these thresholds, suggesting clinically meaningful benefit; in contrast, some gabapentin and nonpharmacologic interventions showed statistically significant but clinically modest improvements.

#### 4.5.2. Measurement Tool Variability and Sensitivity

This UR also highlights the significant variability in QoL instruments used across systematic reviews. While tools such as EORTC QLQ‐C30 offer broad coverage of cancer‐related QoL, others like FACT–H&N and UW‐QOL provide more granular assessment of head and neck‐specific symptoms. Notably, FACT–H&N is designed to capture treatment‐induced toxicities, including mucositis‐related pain and swallowing difficulties, whereas UW‐QOL includes psychosocial constructs like mood and anxiety. In contrast, VASs for pain, although widely used, lack multidimensionality and are poorly suited for tracking complex symptom burden. We recommend the adoption of validated, multidimensional tools such as EORTC QLQ‐C30 or FACT–H&N in future OM‐related trials, ideally complemented by symptom‐specific modules and anchored to established MID values. Standardization would enhance comparability across studies, facilitate meta‐analytical synthesis, and support regulatory and clinical uptake of QoL data. Our findings underscore the urgent need for an international consensus on QoL measurement in OM research, ideally leading to a harmonized core set of instruments and domains. Such consensus—developed through multistakeholder processes including patients—would greatly improve cross‐study comparability and facilitate meta‐analytic synthesis.

#### 4.5.3. PPI

Although no patients were directly involved in the conduct of this UR, its design and outcome focus were informed by published literature emphasizing patient‐reported experiences of OM. Multiple included reviews referenced the significant distress, functional impairment, and emotional burden OM imposes on patients, particularly in head and neck cancer populations. These patient‐centered concerns shaped the prioritization of QoL as the primary outcome and influenced the decision to use tools with proven sensitivity to psychosocial and functional domains. Future URs may benefit from direct patient involvement in defining relevant outcome domains and interpreting intervention impact.

#### 4.5.4. Implications for Clinical Practice and Research

PBMT and honey have the most robust evidence for QoL improvement and should be prioritized in clinical practice [19, 20]. Black mulberry and SAMITAL are promising but require further validation [22, 23]. Chamomile remains controversial, with delivery method and formulation being key factors in efficacy [21].

From a policy perspective, consistent use of validated QoL instruments, registration of systematic review protocols (e.g., PROSPERO), and integration of PROs into OM trials are urgently needed [24, 25]. As demonstrated by Potrich [19] and Zhang [23], domain‐level analyses and adherence to reporting standards enhance the interpretability and clinical utility of findings.

### 4.6. Limitations

This UR is not without limitations. First, we included only English‐language systematic reviews. Second, we relied on summary data without re‐examining primary studies. Third, QoL heterogeneity and missing data on MIDs hinder direct comparisons. Additionally, interventions such as PBMT had standardized protocols, while botanical agents were often used in unregulated forms, raising questions about reproducibility and scalability. First, we limited inclusion to English‐language systematic reviews, which may have excluded relevant evidence synthesized in other languages and introduced language and publication bias, particularly in regions where complementary and botanical interventions are commonly used but less frequently published in English‐language journals.

### 4.7. Future Directions

Future systematic reviews should:

• Prioritize QoL as a primary endpoint with standardized measurement (e.g., EORTC QLQ‐C30, UW‐QOL);
• Define and report MIDs in QoL scores;
• Register protocols and apply PRISMA/AMSTAR 2 standards;
• Include diverse cancer types and patient populations;
• Use longitudinal designs to assess the sustainability of QoL improvements.

We recommend the development of a core outcome set for OM‐related QoL, defining a minimum set of domains, instruments, and reporting standards to be used consistently in future trials and systematic reviews. As reporting of QoL outcomes becomes more standardized, future URs could explore quantitative integration of effect sizes (e.g., meta‐analysis or meta‐regression of summary estimates drawn from high‐quality systematic reviews), thereby strengthening inferences regarding comparative effectiveness and dose–response relationships.

## 5. Conclusion

This UR offers the first comprehensive synthesis of systematic reviews assessing how therapeutic interventions for OM affect QoL in cancer patients. Interventions such as PBMT, honey, SAMITAL, and black mulberry formulations consistently improved QoL domains like pain relief, oral function, and emotional well‐being.

PBMT stood out as the most robustly supported approach, with strong methodological backing and high patient acceptability. Natural agents such as honey and SAMITAL also showed meaningful, accessible benefits. In contrast, interventions like chamomile and nutritional support require further validation regarding formulation and delivery.

Despite positive findings, gaps persist. Many studies lack validated QoL instruments, fail to report MIDs, and offer limited subgroup analysis. QoL outcomes are often secondary, inconsistently measured, and poorly reported.

This review emphasizes the need for patient‐centered research. Future trials should prioritize validated QoL endpoints, symptom‐specific interventions, and patient preferences. Reviews must meet rigorous methodological standards and clearly address clinical relevance and bias.

Integrating effective, noninvasive, and low‐cost interventions like PBMT and honey into clinical guidelines is essential. Personalized OM care, grounded in QoL, should guide future supportive oncology strategies.

## Funding

This research received no specific grant from any funding agency in the public, commercial, or not‐for‐profit sectors.

## Ethics Statement

This review is based exclusively on secondary analysis of published data; therefore, ethical approval and informed consent were not required.

## Consent

The authors have nothing to report.

## Conflicts of Interest

The authors declare no conflicts of interest.

## Supporting Information

Additional supporting information can be found online in the Supporting Information section.

## Supporting information


**Supporting Information 1** Supporting Table 1 (Search Strategy). Supporting Table 1 details the comprehensive and systematic search strategy employed to identify relevant studies for inclusion in this review. The search was conducted across multiple electronic databases, including PubMed/MEDLINE, Scopus, Web of Science, Embase, and the Cochrane Library, to ensure broad coverage of the available literature. The search strategy was structured around three core conceptual domains: (1) OM and related conditions, (2) therapeutic and preventive interventions, including photobiomodulation, pharmacological agents, topical treatments, and oral care protocols, and (3) patient‐centered outcomes, with particular emphasis on quality of life, pain management, oral function, treatment adherence, and validated patient‐reported outcome measures. Controlled vocabulary terms (e.g., MeSH and Emtree terms) were combined with free‐text keywords using Boolean operators to maximize sensitivity and specificity. Filters were applied, when appropriate, to restrict results to systematic reviews, meta‐analyses, and original research articles, depending on the database. No restrictions on publication year were applied during the initial search to capture the full scope of the evidence. This detailed reporting of the search strategy enhances the transparency, reproducibility, and methodological robustness of the review process, in accordance with PRISMA and best practices for evidence synthesis.

**Supporting Information 2** Supporting Table S2 (AMSTAR 2 Assessment). Supporting Table S2 presents the methodological quality assessment of the systematic reviews included in this umbrella review, conducted using the AMSTAR 2 (A MeaSurement Tool to Assess Systematic Reviews 2) instrument. AMSTAR 2 is a validated and widely accepted critical appraisal tool designed to evaluate the methodological rigor of systematic reviews that include randomized controlled trials (RCTs), nonrandomized studies of interventions (NRSI), or both. The table reports the evaluation of each included systematic review across the 16 AMSTAR 2 domains, encompassing key methodological aspects such as the clarity of the research question framed using the PICO components, the comprehensiveness of the literature search strategy, duplicate study selection and data extraction, assessment and consideration of risk of bias, appropriateness of meta‐analytical methods (when applicable), and transparency in reporting funding sources and conflicts of interest. Critical domains, as defined by the AMSTAR 2 guidelines, are explicitly identified and highlighted. Based on the presence or absence of critical and noncritical methodological flaws, an overall confidence rating is assigned to each review (high, moderate, low, or critically low). This structured appraisal allows a transparent interpretation of the strength and reliability of the secondary evidence and supports the cautious integration of findings into the overall synthesis and conclusions of the umbrella review.

## Data Availability

All data analyzed during this work are included in this published article. Any additional data are available on request from the authors.
