# Supplementary material for: Therapeutic Interventions to Manage Oral Mucositis and Their Impact on Quality of Life in Cancer Patients: An Umbrella Review
Source: Pain Res Manag. 2026 Feb 3;2026:3601001. doi: 10.1155/prm/3601001 (PMC12868910; doi:10.1155/prm/3601001)
Supplement: Supplementary file 1 — Supporting Information 1 Supporting Table 1 (Search Strategy). Supporting Table 1 details the comprehensive and systematic search strategy employed to identify relevant studies for inclusion in this review. The search was conducted across multiple electronic databases, including PubMed/MEDLINE, Scopus, Web of Science, Embase, and the Cochrane Library, to ensure broad coverage of the available literature. The search strategy was structured around three core conceptual domains: (1) OM and related conditions, (2) therapeutic and preventive interventions, including photobiomodulation, pharmacological agents, topical treatments, and oral care protocols, and (3) patient‐centered outcomes, with particular emphasis on quality of life, pain management, oral function, treatment adherence, and validated patient‐reported outcome measures. Controlled vocabulary terms (e.g., MeSH and Emtree terms) were combined with free‐text keywords using Boolean operators to maximize sensitivity and specificity. Filters were applied, when appropriate, to restrict results to systematic reviews, meta‐analyses, and original research articles, depending on the database. No restrictions on publication year were applied during the initial search to capture the full scope of the evidence. This detailed reporting of the search strategy enhances the transparency, reproducibility, and methodological robustness of the review process, in accordance with PRISMA and best practices for evidence synthesis. [file PRM-2026-3601001-s001.docx]

**Table Supplementary 1**

| **Database** | **Formulation** | **Filters** |
| --- | --- | --- |
| Pubmed | ((“oral mucositis”[Title/Abstract] OR “mucositis”[Title/Abstract] OR “chemotherapy-induced mucositis”[Title/Abstract] OR  “radiation-induced mucositis”[Title/Abstract] OR “oral complications of cancer therapy”[Title/Abstract] OR  “mucosal toxicity”[Title/Abstract]) OR "Mucositis"[MeSH])  AND  ("photobiomodulation"[Title/Abstract] OR "low-level laser therapy"[Title/Abstract] OR "LLLT"[Title/Abstract] OR "laser therapy"[Title/Abstract] OR  "cryotherapy"[Title/Abstract] OR "oral care"[Title/Abstract] OR "oral hygiene protocols"[Title/Abstract] OR  "mouthwash"[Title/Abstract] OR "mouthwashes"[Title/Abstract] OR "pharmacologic treatment"[Title/Abstract] OR  "pharmacologic agents"[Title/Abstract] OR "topical agents"[Title/Abstract] OR "natural products"[Title/Abstract] OR  "analgesics"[Title/Abstract] OR "chlorhexidine"[Title/Abstract] OR "honey"[Title/Abstract] OR "zinc supplements"[Title/Abstract] OR  "palifermin"[Title/Abstract] OR "amifostine"[Title/Abstract])  AND  ("quality of life"[Title/Abstract] OR "QoL"[Title/Abstract] OR "pain relief"[Title/Abstract] OR "pain management"[Title/Abstract] OR  "pain reduction"[Title/Abstract] OR "oral function"[Title/Abstract] OR "speech function"[Title/Abstract] OR  "swallowing ability"[Title/Abstract] OR "eating difficulties"[Title/Abstract] OR "treatment adherence"[Title/Abstract] OR  "treatment compliance"[Title/Abstract] OR "patient-reported outcomes"[Title/Abstract] OR "functional outcomes"[Title/Abstract] OR  "Oral Health Impact Profile"[Title/Abstract] OR "OHIP"[Title/Abstract] OR "EORTC QLQ-C30"[Title/Abstract] OR  "EORTC QLQ-H&N35"[Title/Abstract] OR "FACT-G"[Title/Abstract] OR "FACT-H&N"[Title/Abstract] OR "UW-QOL"[Title/Abstract] OR  "WHOQOL"[Title/Abstract] OR "SF-36"[Title/Abstract] OR "EQ-5D"[Title/Abstract] OR  "Quality of Life"[MeSH] OR "Pain Management"[MeSH] OR "Pain"[MeSH] OR "Patient Compliance"[MeSH]) | Filters applied: Meta-Analysis, Systematic Review. |
| Scopus | TITLE-ABS-KEY("oral mucositis" OR "mucositis" OR "chemotherapy-induced mucositis" OR  "radiation-induced mucositis" OR "oral complications of cancer therapy" OR "mucosal toxicity")  AND  TITLE-ABS-KEY("photobiomodulation" OR "low-level laser therapy" OR "LLLT" OR "laser therapy" OR  "cryotherapy" OR "oral care" OR "oral hygiene protocols" OR "mouthwash" OR "mouthwashes" OR  "pharmacologic treatment" OR "pharmacologic agents" OR "topical agents" OR "natural products" OR  "analgesics" OR "chlorhexidine" OR "honey" OR "zinc supplements" OR "palifermin" OR "amifostine")  AND  TITLE-ABS-KEY("quality of life" OR "QoL" OR "pain relief" OR "pain management" OR "pain reduction" OR  "oral function" OR "speech function" OR "swallowing ability" OR "eating difficulties" OR  "treatment adherence" OR "treatment compliance" OR "patient-reported outcomes" OR "functional outcomes" OR  "Oral Health Impact Profile" OR "OHIP" OR "EORTC QLQ-C30" OR "EORTC QLQ-H&N35" OR  "FACT-G" OR "FACT-H&N" OR "UW-QOL" OR "WHOQOL" OR "SF-36" OR "EQ-5D") | AND ( LIMIT-TO ( DOCTYPE , "re" ) OR LIMIT-TO ( DOCTYPE , "ar" ) ) |
| WoS | TS=("oral mucositis" OR "mucositis" OR "chemotherapy-induced mucositis" OR  "radiation-induced mucositis" OR "oral complications of cancer therapy" OR "mucosal toxicity")  AND  TS=("photobiomodulation" OR "low-level laser therapy" OR "LLLT" OR "laser therapy" OR  "cryotherapy" OR "oral care" OR "oral hygiene protocols" OR "mouthwash" OR "mouthwashes" OR  "pharmacologic treatment" OR "pharmacologic agents" OR "topical agents" OR "natural products" OR  "analgesics" OR "chlorhexidine" OR "honey" OR "zinc supplements" OR "palifermin" OR "amifostine")  AND  TS=("quality of life" OR "QoL" OR "pain relief" OR "pain management" OR "pain reduction" OR  "oral function" OR "speech function" OR "swallowing ability" OR "eating difficulties" OR  "treatment adherence" OR "treatment compliance" OR "patient-reported outcomes" OR "functional outcomes" OR  "Oral Health Impact Profile" OR "OHIP" OR "EORTC QLQ-C30" OR "EORTC QLQ-H&N35" OR  "FACT-G" OR "FACT-H&N" OR "UW-QOL" OR "WHOQOL" OR "SF-36" OR "EQ-5D") | Refined By:Document Types: Review Article or Article |
| Embase | ('oral mucositis':ab,ti OR 'mucositis':ab,ti OR 'chemotherapy-induced mucositis':ab,ti OR  'radiation-induced mucositis':ab,ti OR 'oral complications of cancer therapy':ab,ti OR 'mucosal toxicity':ab,ti)  AND  ('photobiomodulation':ab,ti OR 'low-level laser therapy':ab,ti OR 'LLLT':ab,ti OR 'laser therapy':ab,ti OR  'cryotherapy':ab,ti OR 'oral care':ab,ti OR 'oral hygiene protocols':ab,ti OR 'mouthwash':ab,ti OR 'mouthwashes':ab,ti OR  'pharmacologic treatment':ab,ti OR 'pharmacologic agents':ab,ti OR 'topical agents':ab,ti OR 'natural products':ab,ti OR  'analgesics':ab,ti OR 'chlorhexidine':ab,ti OR 'honey':ab,ti OR 'zinc supplements':ab,ti OR  'palifermin':ab,ti OR 'amifostine':ab,ti)  AND  ('quality of life':ab,ti OR 'QoL':ab,ti OR 'pain relief':ab,ti OR 'pain management':ab,ti OR 'pain reduction':ab,ti OR  'oral function':ab,ti OR 'speech function':ab,ti OR 'swallowing ability':ab,ti OR 'eating difficulties':ab,ti OR  'treatment adherence':ab,ti OR 'treatment compliance':ab,ti OR 'patient-reported outcomes':ab,ti OR 'functional outcomes':ab,ti OR  'Oral Health Impact Profile':ab,ti OR 'OHIP':ab,ti OR 'EORTC QLQ-C30':ab,ti OR 'EORTC QLQ-H&N35':ab,ti OR  'FACT-G':ab,ti OR 'FACT-H&N':ab,ti OR 'UW-QOL':ab,ti OR 'WHOQOL':ab,ti OR 'SF-36':ab,ti OR 'EQ-5D':ab,ti) |  |
| Cochrane Library | ("oral mucositis":ti,ab,kw OR "mucositis":ti,ab,kw OR "chemotherapy-induced mucositis":ti,ab,kw OR  "radiation-induced mucositis":ti,ab,kw OR "oral complications of cancer therapy":ti,ab,kw OR "mucosal toxicity":ti,ab,kw)  AND  ("photobiomodulation":ti,ab,kw OR "low-level laser therapy":ti,ab,kw OR "LLLT":ti,ab,kw OR "laser therapy":ti,ab,kw OR  "cryotherapy":ti,ab,kw OR "oral care":ti,ab,kw OR "oral hygiene protocols":ti,ab,kw OR "mouthwash":ti,ab,kw OR "mouthwashes":ti,ab,kw OR  "pharmacologic treatment":ti,ab,kw OR "pharmacologic agents":ti,ab,kw OR "topical agents":ti,ab,kw OR "natural products":ti,ab,kw OR  "analgesics":ti,ab,kw OR "chlorhexidine":ti,ab,kw OR "honey":ti,ab,kw OR "zinc supplements":ti,ab,kw OR  "palifermin":ti,ab,kw OR "amifostine":ti,ab,kw)  AND  ("quality of life":ti,ab,kw OR "QoL":ti,ab,kw OR "pain relief":ti,ab,kw OR "pain management":ti,ab,kw OR  "pain reduction":ti,ab,kw OR "oral function":ti,ab,kw OR "speech function":ti,ab,kw OR "swallowing ability":ti,ab,kw OR  "eating difficulties":ti,ab,kw OR "treatment adherence":ti,ab,kw OR "treatment compliance":ti,ab,kw OR  "patient-reported outcomes":ti,ab,kw OR "functional outcomes":ti,ab,kw OR  "Oral Health Impact Profile":ti,ab,kw OR "OHIP":ti,ab,kw OR "EORTC QLQ-C30":ti,ab,kw OR "EORTC QLQ-H&N35":ti,ab,kw OR  "FACT-G":ti,ab,kw OR "FACT-H&N":ti,ab,kw OR "UW-QOL":ti,ab,kw OR "WHOQOL":ti,ab,kw OR "SF-36":ti,ab,kw OR "EQ-5D":ti,ab,kw) | |
