# Supplementary material for: Therapeutic Interventions to Manage Oral Mucositis and Their Impact on Quality of Life in Cancer Patients: An Umbrella Review
Source: Pain Res Manag. 2026 Feb 3;2026:3601001. doi: 10.1155/prm/3601001 (PMC12868910; doi:10.1155/prm/3601001)
Supplement: Supplementary file 2 — Supporting Information 2 Supporting Table S2 (AMSTAR 2 Assessment). Supporting Table S2 presents the methodological quality assessment of the systematic reviews included in this umbrella review, conducted using the AMSTAR 2 (A MeaSurement Tool to Assess Systematic Reviews 2) instrument. AMSTAR 2 is a validated and widely accepted critical appraisal tool designed to evaluate the methodological rigor of systematic reviews that include randomized controlled trials (RCTs), nonrandomized studies of interventions (NRSI), or both. The table reports the evaluation of each included systematic review across the 16 AMSTAR 2 domains, encompassing key methodological aspects such as the clarity of the research question framed using the PICO components, the comprehensiveness of the literature search strategy, duplicate study selection and data extraction, assessment and consideration of risk of bias, appropriateness of meta‐analytical methods (when applicable), and transparency in reporting funding sources and conflicts of interest. Critical domains, as defined by the AMSTAR 2 guidelines, are explicitly identified and highlighted. Based on the presence or absence of critical and noncritical methodological flaws, an overall confidence rating is assigned to each review (high, moderate, low, or critically low). This structured appraisal allows a transparent interpretation of the strength and reliability of the secondary evidence and supports the cautious integration of findings into the overall synthesis and conclusions of the umbrella review. [file PRM-2026-3601001-s002.docx]

**Supplementary Table S2. AMSTAR 2 methodological quality assessment**

| **Reviews** | **1. PICO components** | **2*. Pre-established protocol** | **3. Explanation of included studies’ design** | **4*. Comprehensive search strategy** | **5. Duplicate study selection** | **6. Duplicate data extraction** | **7*. List of excluded studies and justification** | **8. Description of included studies** | **9*. Assessment of RoB in included studies** | **10. Funding sources** | **11*. Use of appropriate statistical methods** | **12. RoB impact on synthesized results** | **13*. Results interpretation with RoB reference** | **14. Heterogeneity explanation** | **15*. Publication / small study bias investigation** | **16. Conflict of interest declaration** | **Overall confidence** |
| --- | --- | --- | --- | --- | --- | --- | --- | --- | --- | --- | --- | --- | --- | --- | --- | --- | --- |
| Liu 2019 | Yes | Yes | Yes | Yes | Yes | Yes | No | Yes | Yes | No | Yes | Yes | Yes | Yes | Yes | Yes | Low |
| Jin 2023 | Yes | Yes | Yes | Yes | Yes | Yes | No | Yes | Yes | Yes | Yes | Yes | Yes | Yes | Yes | Yes | Low |
| Maleki 2023 | Yes | Yes | Yes | Yes | Yes | Yes | Yes | Yes | Yes | Yes | NA | Yes | Yes | Yes | No | Yes | Low |
| Sánchez-Martos 2023 | Yes | No | Yes | Partial Yes | Yes | Yes | No | Yes | No | NA | NA | NA | NA | Yes | No | Yes | Critically Low |
| Potrich 2024 | Yes | Yes | Yes | Yes | Yes | Yes | Yes | Yes | Yes | NA | NA | Yes | Yes | Yes | No | Yes | Low |
| Sindhe 2024 | Yes | Yes | Yes | Yes | Yes | Yes | Yes | Yes | Yes | Yes | NA | Yes | Yes | Yes | No | Yes | Low |
| Zhang 2024 | Yes | Yes | Yes | Yes | Yes | Yes | No | Yes | Yes | No | NA | Yes | Yes | Yes | No | Yes | Critically Low |
| Baig 2025 | Yes | Yes | Yes | Yes | Yes | Yes | Yes | Yes | Yes | No | NA | NA | NA | Yes | No | Yes | Low |

**Supplementary Table S2 AMSTAR 2**

A MeaSurement Tool to Assess Systematic Reviews 2 PICO, participant, intervention, comparison, outcome; RoB, Risk of bias.

*Asterisk indicates a critical item (domain).

A Possible responses: Yes/No.

B Possible responses: Yes/Partial Yes/No. A 'Partial Yes' response is evaluated positively to the overall confidence rating.

C Item response depends on separate assessment of randomized controlled trials (RCTs) and non-randomized studies of healthcare interventions (NRSI) in the review.

D Possible responses: Yes/No/No MA. A 'No MA' response stands for 'no meta-analysis conducted' and does not affect the overall confidence rating.

E Critically Low/Low/Moderate/High confidence in the results of the review. Critically Low: more than one critical flaw with or without non-critical weaknesses, Low: One critical flaw with or without non-critical weaknesses, Moderate: No critical flaws but more than one non-critical weakness, High: No flaws at all or one non-critical weakness.
